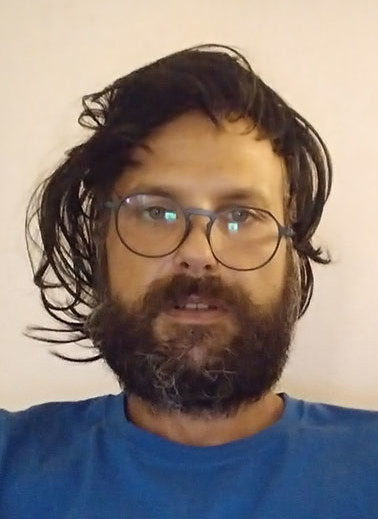# Surface Chemistry Impact on the Light Absorption by Colloidal Quantum Dots

**DOI:** 10.1002/chem.202102168

**Published:** 2021-09-13

**Authors:** Carlo Giansante

**Affiliations:** ^1^ Carlo Giansante CNR NANOTEC, Istituto di Nanotecnologia Via Monteroni 73100 Lecce Italy

**Keywords:** Colloidal Nanocrystals, Ligands, Light Absorption, Quantum Dots, Surface Chemistry

## Abstract

At the size scale at which quantum confinement effects arise in inorganic semiconductors, the materials’ surface‐to‐volume ratio is intrinsically high. This consideration sets surface chemistry as a powerful tool to exert further control on the electronic structure of the inorganic semiconductors. Among the materials that experience the quantum confinement regime, those prepared via colloidal synthetic procedures (the colloidal quantum dots – and wires and wells, too –) are prone to undergo surface reactions in the solution phase and thus represent an ideal framework to study the ensemble impact of surface chemistry on the materials’ electronic structure. It is here discussed such an impact at the ground state by using the absorption spectrum of the colloidal quantum dots as a descriptor. The experiments show that the chemical species (the ligands) at the colloidal quantum dot surface induce changes to the optical band gap, the absorption coefficient at all wavelengths, and the ionization potential. These evidences point to a description of the colloidal quantum dot (the ligand/core adduct) as an indecomposable species, in which the orbitals localized on the ligands and the core mix in each other's electric field. This description goes beyond conventional models that conceive the ligands on the basis of pure electrostatic arguments (i. e., either as a dielectric shell or as electric dipoles) or as a mere potential energy barrier at the core boundaries.

## Graphical abstract

Ligands at the surface of colloidal quantum dots are intrinsically electronically coupled to the inorganic semiconductor core thus affecting the overall ground state electronic structure and, consequently, the light absorption properties.

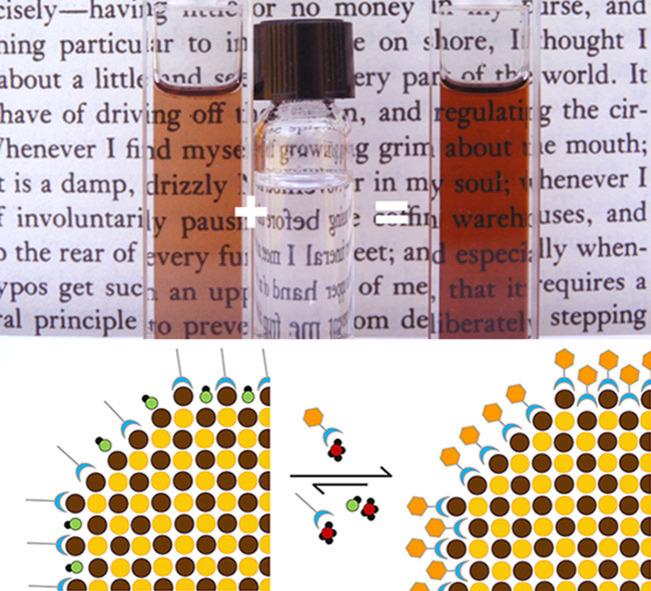

## Introduction

Quantum dots (QDs) are inorganic semiconductor materials with size, in all the three dimensions, that approaches the most probable distance between the electron and hole probability distributions in an exciton, which is of the order of 10^−9^–10^−8^ meters depending on the materials’ band structure. At this size scale, quantum confinement effects arise and the QDs show a size dependent electronic structure.^[1]^ At this size scale, the surface‐to‐volume ratio is intrinsically high and the chemical species at the QD surface may further affect the electronic structure.[^2^, ^3^] Among other methods, the QDs can be prepared by chemical synthesis in the solution phase yielding dispersions that are colloidally stable, mainly due to the steric repulsion between the hydrophobic tails of the amphiphilic ligands that coordinate the QD surface.[^4^, ^5^] The as‐synthesized colloidal QDs are amenable to undergo surface chemical reactions in the solution phase, thus enabling the use of common spectroscopic techniques to study nanoscopic surfaces and their ensemble effects on the QD electronic structure.[^6^, ^7^] To this aim, the coordination of the QD surface by chemical species that replace the ligands coming from the synthetic procedures is of capital importance and its reliability depends on a rigorous description of the QDs at the atomic level.[^8^, ^9^]

On this basis, it is here discussed how surface chemistry impacts the QD electronic structure at the ground state, thus affecting the optical absorption properties. Light absorption by colloidal QDs is diagnostic of the ground state electronic structure and provides information on the QD size, size distribution, and concentration in solution. Light absorption is also the event that presides over the light energy conversion processes that exploit the colloidal QDs both in commercial goods, as luminophores for display and illumination technologies, and in academic research, as photovoltaic materials and photocatalytic substrates. The control of the QD surface is of paramount importance in such applications, aiming at the passivation of surface mid gap states that may trap the photogenerated charge carriers.^[10]^ While efficient photoluminescence mainly relies on the growth of an epitaxial inorganic shell onto the emissive QD core,^[11]^ photovoltaic and photodetection applications are based on QD arrays and atomic/small molecule ligands are commonly employed to ensure efficient inter‐QD charge transport;[^12^, ^13^] more subtly, homogeneous photocatalysis requires a QD surface chemistry that guarantees both the access to the substrate and the resistance to corrosion, while preserving the colloidal stability.^[14]^

This widespread recourse to ligand exchange procedures has led to disclose, somewhat serendipitously, the surface chemistry impact on the QD ground state electronic structure. Indeed, post‐synthesis surface chemistry modification has been shown to induce large changes in the band edge energies,^[15]^ the optical band gap,^[16]^ and the absorption coefficient at all energies,^[17]^ which occur concomitantly,^[18]^ even though, in some cases, their magnitude may let one phenomenon prevail on the others. It is here discussed the origin of such ligand‐induced effects, which is ascribed to the mixing of orbitals localized on the ligands and the core in each other's electric field. This notion goes beyond the elegantly simple models that conceive ligands as electric dipoles shifting the core band energies,[^15^, ^19^] as potential barriers allowing the delocalization of the exciton outside the core,^[20]^ and as dielectric shells perturbing the electric field experienced by the core.[^21^, ^22^] An explanation to the experimental observations cannot even be conceived on the basis of these simple models, which treat the inorganic core coordinated by the ligands as a mere superposition of the ligand and core components and the ligand/core interface as abrupt. While the band energy shifting cannot be directly measured by optical absorption spectroscopy, the changes in the optical band gap and the absorption coefficients can instead be easily measured in cuvette by common spectrophotometers. Therefore, these two ligand‐induced phenomena are extensively discussed hereafter, although the concomitant band energy shifting is mentioned when appropriate.

## The Absorption Spectrum of the Colloidal QDs

**Figure 1:**
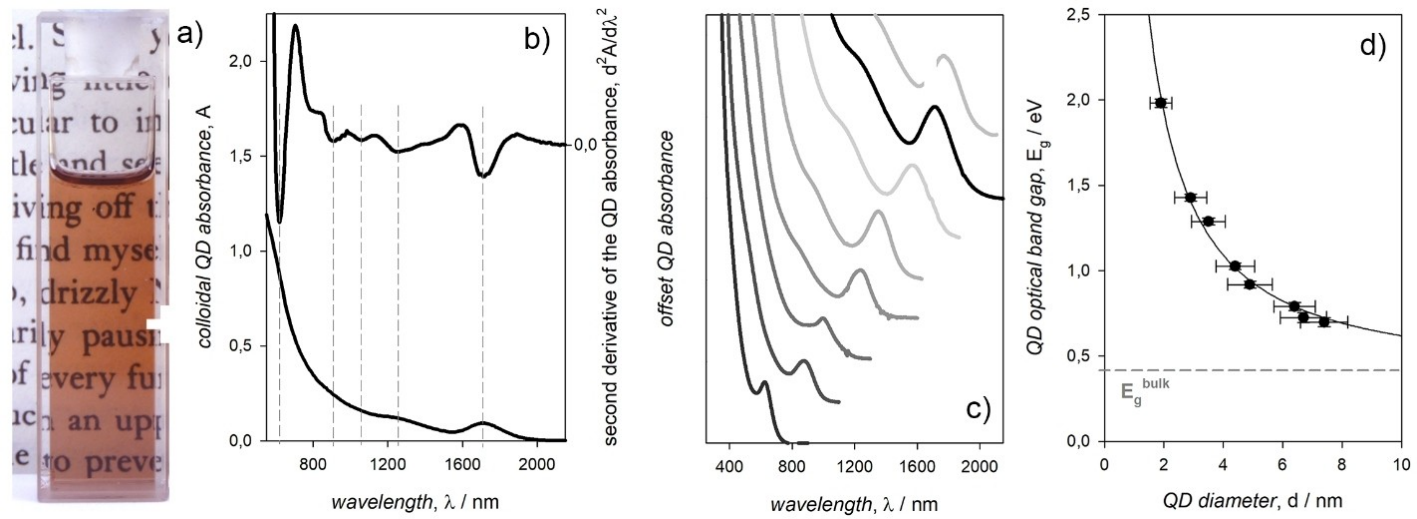
a) Daylight picture of a quartz cuvette containing a tetrachloroethylene dispersion of as‐synthesized and purified colloidal PbS QDs. b) Absorption spectrum of such a QD dispersion and its second derivative. c) Offset, normalized absorption spectra of colloidal PbS QDs with different sizes. d) Sizing curve relating the optical band gap, E_g_, of colloidal PbS QDs with their diameter measured by statistical analysis of TEM images. Adapted with permission from ref. [26]; copyright 2018, American Chemical Society.

As‐synthesized colloidal QDs are highly dispersible in solvents leading to optically transparent samples, in which the attenuation of the incident light can be considered as entirely due to the QD absorption, being the scattering negligible (Figure 1a). The absorption spectrum of the colloidal QDs is characterized by a sharp peak at low energies (Figure 1b), which corresponds to the lowest energy exciton and defines the QD optical band gap. Second derivative analysis of the absorption spectrum is useful to identify higher energy excitons (Figure 1b). Due to quantum confinement, the energy of the first exciton peak is size dependent (Figure 1c) and it can be used to estimate the QD diameter by means of sizing curves (Figure 1d) that were determined through a combination of elemental analysis, electron microscopy imaging, and optical absorption measurements. The case of PbS is instructive in this regard since the reliable colloidal synthesis of the QDs, the narrow band gap and the large exciton Bohr radius of the bulk material, including the effect of the small charge carrier effective masses, allow the observation of sharp absorption peaks shifting across a wide spectral range corresponding to QDs with measurably different sizes. Indeed, different studies reported similar sizing curves for colloidal PbS QDs.[^23^, ^24^, ^25^, ^26^] Analogously, sizing curves were proposed for Cd chalcogenides[^27^, ^28^] and for lead halide perovskites.^[29]^

**Figure 2:**
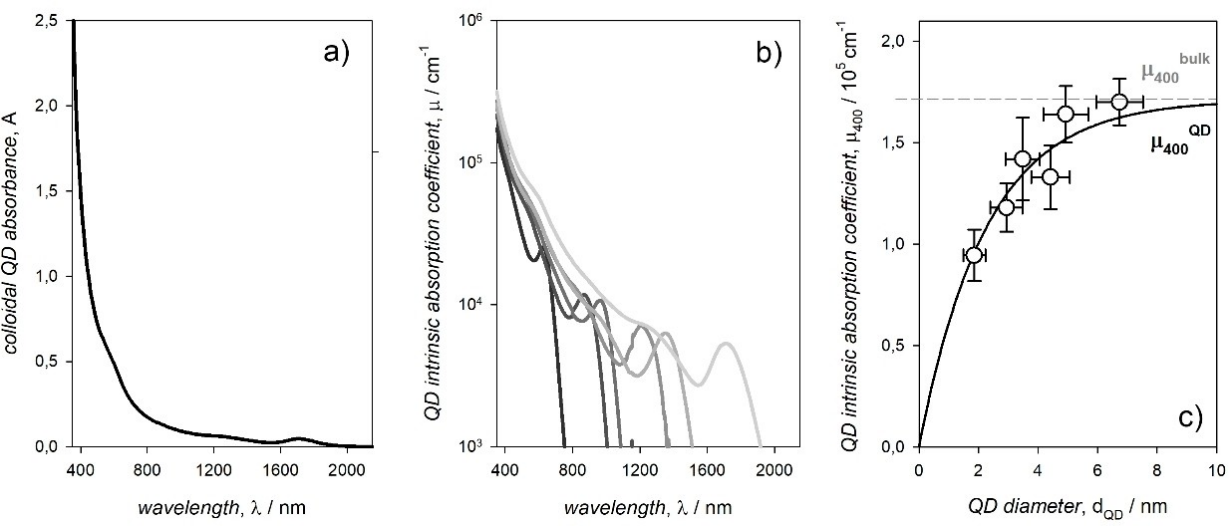
a) Absorption spectrum of a tetrachloroethylene dispersion of colloidal PbS QDs. b) Intrinsic absorption coefficients, μ, of colloidal PbS QDs with different sizes. c) Sizing curve relating the intrinsic absorption coefficient at 400 nm, μ_400_, of colloidal PbS QDs with their diameter measured by statistical analysis of TEM images. Adapted with permission from ref. [31]; copyright 2017, American Chemical Society.

At high energies, the absorption spectrum of the colloidal QDs shows a rise (Figure 2a), which reflects the high density of states at energies far from the band edges that approaches a continuum. Indeed, quantum confinement diminishes, until vanishing at energies sufficiently far from the band gap. In the absence of scattering, the intrinsic, thickness independent absorption coefficient of the colloidal QDs at high energies is expected to match that of the corresponding bulk material, rescaled for the dielectric confinement effect exerted by the surrounding ligands and the solvent. Therefore, the intrinsic absorption coefficient of the colloidal QDs at energies sufficiently far from the band gap can be considered as size independent.[^6^, ^22^, ^30^] In the case of PbS QDs, the energy at which quantum confinement ceases is estimated as of about 3.1 eV, corresponding to 400 nm (Figure 2b). However, a deviation for PbS QDs with diameter below about 4 nm was observed (Figure 2c).^[31]^ Such a deviation for small, strongly quantum confined QDs is not surprising and does not affect the validity of considering the quantum confinement as negligible at energies far from the band gap; it simply implies that far means at energies higher than 3.1 eV (wavelengths below 400 nm). Except for the correction required to account for the deviation, the molar absorption coefficient linearly scales with the QD volume thus permitting, by using the Lambert‐Beer law, a ready spectrophotometric determination of the concentration of QD dispersions. Except for such a correction, a high density of states implies that the error of estimating the QD concentration by absorbance measurements at 400 nm is lower compared to the error of measurements at the first exciton peak, whose absorption coefficient is intrinsically affected by the sample polydispersity (quantum confinement increases the oscillator strength of the electronic transitions, as it can be observed for the PbS QD first exciton peaks in Figure 2b, i. e. the smaller the QD the larger the absorption coefficient at the first exciton peak). Except for the correction, this method to estimate the QD concentration was conveniently applied to Cd chalcogenides,[^22^, ^28^, ^30^, ^32^, ^33^] In pnictides,[^34^, ^35^] Pb chalcogenides,[^28^, ^36^] and Pb halide perovskites.^[37]^

## Ligand Exchange at the QD Surface

Post‐synthesis surface chemistry modification reactions are widely pursued in the colloidal QD science and bonding at the QD surface is being consistently discussed in the scientific literature.[^5^, ^38^] Here, however, bonding at the QD surface is not explicitly discussed; it is instead mentioned the methodology that may be usedto perform ligand exchange reactions to reliably evaluate the surface chemistry effects on the QD light absorption. To this aim, it is preferable to carry out the ligand exchange reactions in the solution phase by adding aliquots of the replacing ligand solutions to the QD dispersions in the same solvent.[^17^, ^39^] This guarantees both a complete access to the surface of the free standing QDs and the control of the QD concentration that simply rescales by the added volume (which can be neglected by employing highly concentrated ligand solutions). Such a control of the QD concentration is prevented when gel permeation chromatography is used to perform ligand exchange reactions, which, however, has the advantage of yielding ligand exchanged QDs without the need for further purification.^[40]^ If the replacing ligands are insoluble in the solvent originally used to disperse the QDs, a phase transfer reaction may be performed; however, this method is prone to alter the QD concentration especially when it requires filtering of the ligand exchanged QD dispersions,^[20]^ thus hindering quantitative comparisons before and after the ligand exchange reactions. In addition, if the ligand exchanged QDs are hardly dispersible, the surface reactions may be performed in the solid state by exposing the QD films to a solution of the replacing ligands;^[41]^ albeit effective, this method does not allow to discriminate the eventual ligand‐induced effects from the inter‐QD interactions that may take place in densely packed QD solids.^[18]^

**Figure 3:**
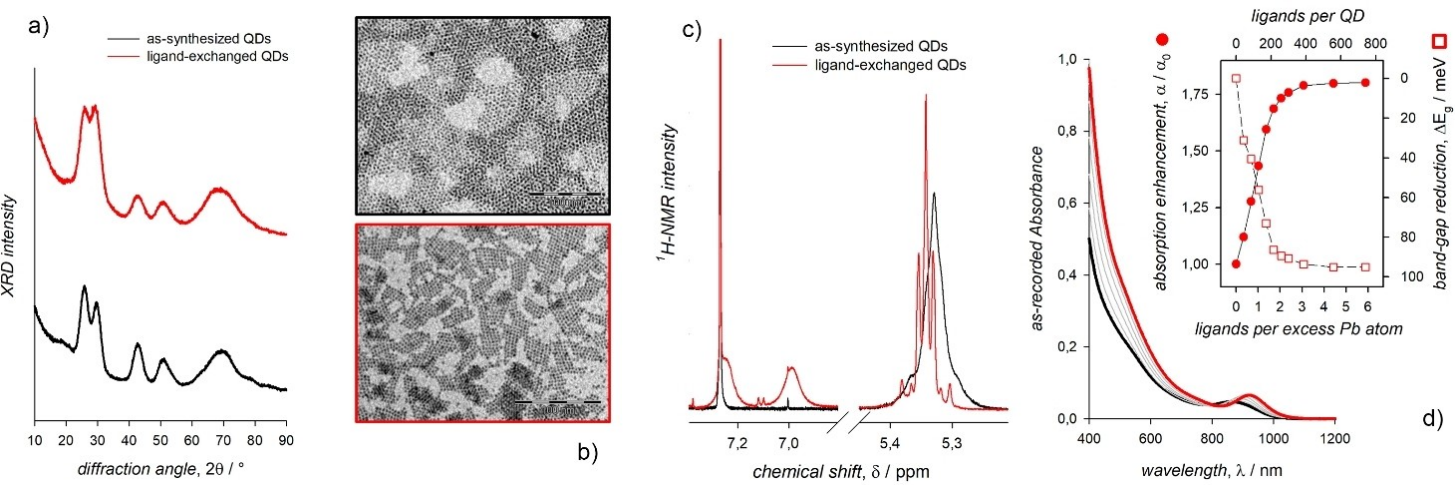
a) XRD patterns of colloidal PbS QDs coordinated by oleate ligands (black line) and of the same QDs upon exchange for *p*‐methylbenzenethiolate ligands (red line). b) TEM images of such as‐synthesized (black line) and ligand‐exchanged (red line) QDs. c) ^1^H‐NMR spectra in dichloromethane‐d2 of the same as‐synthesized (black line) and ligand‐exchanged (red line) QDs omitting spectral regions other than those of the aryl and vinyl protons. d) Absorption spectra in dichloromethane of as‐synthesized PbS QDs (black line) upon addition of *p*‐methylbenzenethiolate as triethylammonium salt; the inset shows a plot of the ligand‐induced spectral changes. Adapted with permission from refs. [17,26]; copyright 2013 and 2018, American Chemical Society.

X‐ray diffraction patterns and electron microscopy imaging can provide evidence that the QD structure and morphology, respectively, do not significantly alter upon ligand exchange (Figures 3a and 3b). Indeed, ligand‐induced structural and morphological transformations are common to lead halide perovskite nanocrystals,[^42^, ^43^] whereas ligand‐induced strain may largely contribute to change the optical properties of the highly anisotropic quantum wires and wells.[^44^, ^45^] The solution phase ligand exchange reactions can be conveniently monitored by NMR spectroscopy probing those resonances characteristic of the replaced and the replacing ligands. Ligands coordinated to the QDs in properly purified samples usually show broadened, downfield shifted resonances compared to those of the same unbound ligands, thus consenting a quantitative evaluation of the effective displacement of the ligands coming from the synthetic procedure.^[7]^ As a example, the vinylene resonance of the oleyl moieties of some of the most common ligands that coordinate the surface of the as‐synthesized QDs (e. g., oleic acid and oleylamine) significantly sharpen and shift upfield upon displacement from the QD surface (Figure 3b). Analogously, broad resonances of the replacing ligands appear in the NMR spectrum of properly ligand exchanged QDs (as shown for the aryl protons of *p*‐methylbenzenethiol in Figure 3b).^[26]^ NMR titration experiments and the use of an inert, internal standard allow the quantitative determination of the amount of replacing ligands necessary to displace the original ligands and the estimation of binding constants.[^46^, ^47^, ^48^, ^49^] These results can be related to the changes observed in the QD absorption spectra, thus permitting to obtain thermodynamic information on the ligand exchange process also by spectrophotometric titration experiments.[^17^, ^18^, ^26^, ^31^, ^50^] For the effective design of ligand exchange reactions and the evaluation of their outcomes, an accurate description of the QDs and their relevant facets based on morphological, structural, and compositional analyses is fundamental. Among other experimental observations that advanced an atomic level description of the colloidal QDs, it is worth mentioning the demonstration of the non‐stoichiometric, metal rich composition of metal chalcogenide QDs,^[36]^ the displacement of metal complexes from the QD surface induced by two‐electron donor ligands (such as amines),^[46]^ and the intrinsic lability of the (metal‐)organic ligands of both metal chalcogenide[^51^, ^52^] and lead halide perovskite QDs.^[53]^ On these bases, the ligand‐induced effects on the light absorption by colloidal QDs can be reliably evaluated. Such an evaluation can take advantage from the use of comprehensive libraries of ligands, as discussed elsewhere.^[3]^

## Ligand‐induced Effects on the QD Optical Band Gap

**Figure 4:**
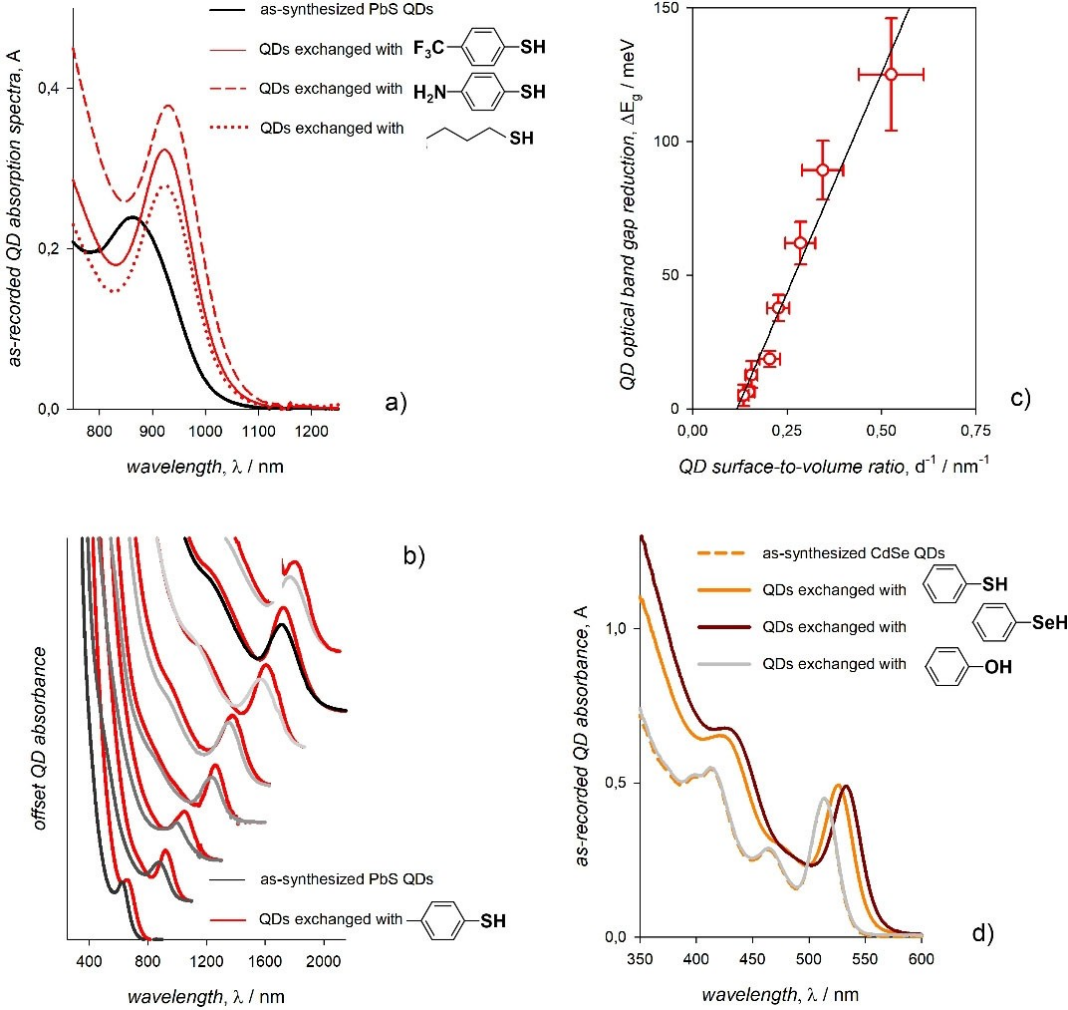
a) Absorption spectra of as‐synthesized PbS QDs (black line) upon addition of arylthiols with electron‐donor (solid red line) and acceptor (dashed red line) substituents and of an alkylthiol (dotted red line). b) Absorption spectra of colloidal PbS QDs with different diameters upon addition of *p*‐methylbenzenethiolate as triethylammonium salt (red lines). c) Plot of the optical band gap reduction induced by *p*‐methylbenzenethiolate ligands as a function of the PbS QD surface‐to‐volume ratio. Adapted with permission from ref. [26]; copyright 2018, American Chemical Society. d) Absorption spectra of as‐synthesized CdSe QDs (dashed orange line) upon addition of phenylchalcogenols, in which the chalcogen atom is O (grey line), S (orange line), or Se (dark red line). Adapted with permission from ref. [59]; copyright 2019, Royal Society of Chemistry.

A narrowing of the optical band gap is often observed when exchanging the metal chalcogenide QD surface for ligands that bind via chalcogen atoms. An early study reported a red shift of the first exciton peak of CdSe QDs induced by alkylthiol ligands as large as few tens of meV.^[16]^ Although such an experimental observation was not thoroughly explained, this study demonstrated that the bathochromic shift was actually induced by the ligands themselves and not by the inter‐QD electronic coupling as commonly perceived at that time. Later, a red shift of several hundred meV induced by phenyldithiocarbamate derivatives was reported for the first exciton of CdSe QDs and, afterwards, also of CdS and PbS QDs.[^20^, ^54^] These red shifts were attributed to the energy level alignment between the ligands’ highest occupied orbitals and the core's valence band edge, which lowers the potential barrier at the core boundaries thus enabling the delocalization of the first exciton on the ligand shell; this delocalization was calculated within the particle‐in‐a‐box analogy upon assuming that the effective hole mass of the ligand shell equals that of the CdSe core. The presence of electron‐donor and acceptor substituents was used to shift the energy levels of the phenyldithiocarbamate ligands and consequently the extent of the exciton delocalization, although this required to assume that the alignment was with a region of the valence band with a high density of states rather than with the valence band edge.^[55]^ This result was not confirmed by other theoretical and experimental studies that also raised doubts on the chemical stability of dithiocarbamates.[^56^, ^57^, ^58^] This result was not confirmed by the experimental evidences obtained using arylthiol ligands substituted with electron‐donating and electron‐withdrawing groups;^[18]^ indeed, the red shift of the first exciton of CdS, CdSe, and PbS QDs induced by benzenethiols was unaffected by the presence in para position of an amino group or a trifluoromethyl group (Figure 4a).[^18^, ^26^, ^59^] An increase of the absorption coefficient at the first exciton peak was also observed, which is opposed to the expectations for a mere exciton delocalization. In addition, alkylthiol ligands induced the same red shift of arylthiols for CdS and PbS (not of CdSe) QD first exciton (Figure 4a), thus pointing to an effect mainly related to the sulfur binding atom of the thiol ligands. This effect is size dependent (Figure 4b): the larger the QD, the smaller the ligand‐induced red shift with an almost linear dependence on the QD surface‐to‐volume ratio (Figure 4c).[^26^, ^59^] Further evidence of the role of the ligand binding group in red shifting the first exciton peak was achieved by using ligands with the same pendant moiety and different binding groups namely phenol, thiophenol, and selenophenol. Such phenylchalcogenols induced a red shift of the CdSe QD first exciton that is larger for ligands with a lower ionization potential (Figure 4d).^[59]^ This observation is similar to what had been previously observed for CdSe QDs coordinated by diphenyldichalcogenide ligands.^[60]^ Overall, these findings were enabled by the above mentioned in‐situ exchange of ligands from a comprehensive library.

**Figure 5:**
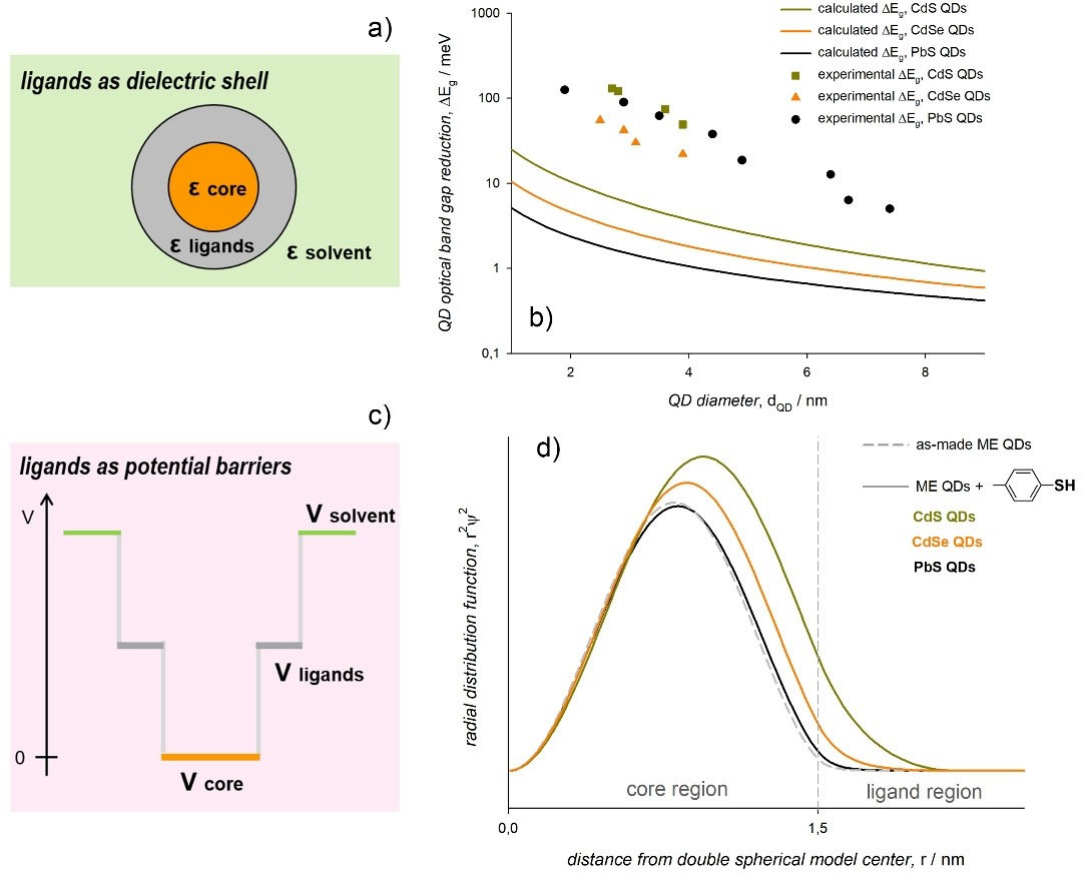
a) Sketch of the dielectric representation of a colloidal QD. b) Size dependent optical band gap reduction, ΔE_g_, induced by p‐methylbenzenethiolate ligands on CdS (dark yellow), CdSe (orange), and PbS (black) QDs that was experimentally observed (symbols) and calculated as a mere dielectric confinement effect (lines). c) Sketch of the colloidal QD representation as a double spherical finite potential well. d) Calculated leakage of the hole wavefunction of CdS (dark yellow), CdSe (orange), and PbS (black) QDs in the *p*‐methylbenzenethiolate ligand shell upon imposing that V_core_=V_ligands_ and m*_h,ligands_=m_e_. Adapted with permission from ref. [59]; copyright 2019, Royal Society of Chemistry.

The red shift was attributed to the *n*p orbitals of the chalcogen binding group that contribute to the valence band edge of the metal chalcogenide QDs, which is mainly constituted by the *n*p orbitals of the chalcogen component of the inorganic core.[^18^, ^26^, ^59^] This explanation could not have been formulated by describing the ligands as either a dielectric shell or as a potential energy barrier. When the ligands are conceived as a dielectric shell (Figure 5a), upon assuming a two‐state, nearly‐free charge carrier model within the framework of the effective mass approximation,^[61]^ the ligands may induce a polarization effect that can be described as a correction to the energy of the QD first exciton.[^22^, ^62^, ^63^] Within this model, the predicted ligand‐induced, size‐dependent reduction of the QD optical band gap is much smaller than that observed experimentally (Figure 5b).[^26^, ^59^] The predicted reduction of the QD optical band gap mainly depends on the dielectric mismatch between the ligand shell and the inorganic core, in agreement with the values of the high‐frequency dielectric constants of the corresponding bulk materials. When the ligands are conceived as a potential energy barrier (Figure 5c), within the effective mass approximation and using the particle in a double spherical finite potential well model, the probability of exciton delocalization on the ligand shell can be regarded as a first order perturbation of the ground state energy.[^61^, ^64^, ^65^, ^66^] Upon assuming that the ligand exchange completely abates the potential energy barrier at the core boundaries, the calculated leakage of the hole wavefunction in the ligand shell contrasts with the experimental observations for QDs with a 3 nm diameter, especially PbS (Figure 5d).[^26^, ^59^] The predicted hole delocalization largely depends on the effective mass mismatch between the ligand shell (the hole effective mass of the ligand region was imposed as the rest mass of an electron, which is a rather fair, although rough assumption)^[67]^ and the inorganic core, in agreement with the values of the hole effective masses of the corresponding bulk materials.

## Ligand‐induced Effects on the QD Absorption Coefficients

**Figure 6:**
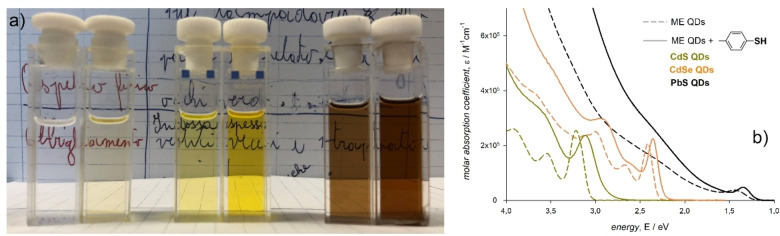
a) Daylight picture of dichloromethane dispersions of colloidal CdS (left), CdSe (center), and PbS (right) QDs before and after the addition of p‐methylbenzenethiolate as triethylammonium salt. b) Molar absorption coefficients of the corresponding QD dispersions. Adapted with permission from ref. [59]; copyright 2019, Royal Society of Chemistry.

The hereinabove discussed reduction of the metal chalcogenide QD optical band gap induced by arylthiol ligands is accompanied by an increase of the absorption coefficient at all wavelengths that can be observed by naked eyes (Figure 6a).[^17^, ^18^, ^26^, ^31^, ^59^] This evidence is only possible when exchanging the ligands in the solution phase without further manipulations. Although such ligands are colorless and only absorb in the UV spectral region, they induce an enhancement of the QD optical absorption across the Vis‐NIR spectral range (Figure 6b).

**Figure 7:**
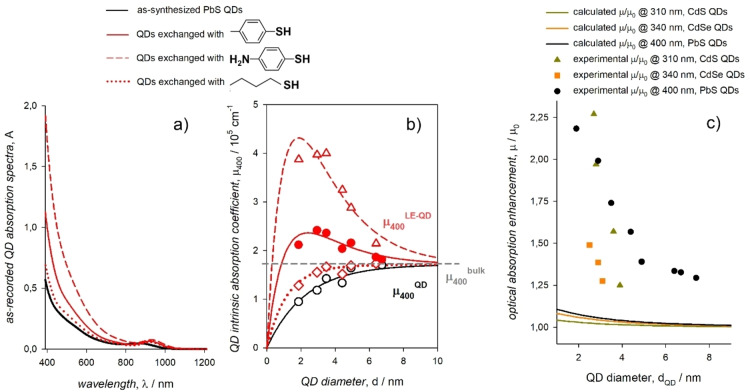
a) Absorption spectra of as‐synthesized PbS QDs (black line) upon addition of arylthiols with methyl (solid red line) and amino (dashed red line) substituents and of an alkylthiol (dotted red line). b) Size dependent plots of the intrinsic absorption coefficient at 400 nm induced by such ligands as a function of the PbS QD diameter. Reproduced from ref. 31 with the permission of the ACS. c) Size dependent optical absorption enhancement at the presumed bulk‐like wavelength, μ/μ_0_, induced by p‐methylbenzenethiolate ligands on CdS (dark yellow), CdSe (orange), and PbS (black) QDs that was experimentally observed (symbols) and calculated within the framework of the Maxwell Garnett effective medium theory (lines). Adapted with permission from ref. [59]; copyright 2019, Royal Society of Chemistry.

Contrarily to the ligand‐induced reduction of the QD optical band gap, in which the ligand effect is mainly due to the binding group, the broadband enhancement of the QD optical absorption is largely dependent on the ligand pendant moiety. Indeed, the absorption enhancement is large (up to 300 % compared to the as‐synthesized QDs) for aromatic thiols, while it is minor (of about 10 %) for aliphatic thiols (Figure 7a). This evidence was quantitatively reproduced by an independent study, which also demonstrated a broadband enhancement of PbS QD absorption by a library of cinnamate derivatives.^[68]^ Such a study suggested that the extent of the broadband absorption enhancement may be related to the energy distance between the highest occupied and the lowest unoccupied orbitals of the ligands themselves. In analogy to the ligand‐induced reduction of the QD optical band gap, the absorption enhancement is size dependent (Figure 7b).[^18^, ^31^, ^59^] This size dependence was evaluated at the wavelength expected to show bulk‐like intrinsic absorption coefficients, which is 400 nm for PbS QDs, albeit for the previously mentioned deviation for small QDs.^[31]^ At this wavelength, it is possible to compare the experimental ligand‐induced QD absorption enhancement with that predicted by the local field approximation resulting from the Maxwell Garnett effective medium theory, in which the ligand shell contributes to the dielectric screening of the external electric field.[^6^, ^69^, ^70^, ^71^] Within this model, the predicted ligand‐induced size‐dependent QD absorption enhancement at wavelengths with expected bulk‐like behavior is much smaller than that observed experimentally (Figure 7c). The predicted QD optical absorption enhancement mainly depends on the dielectric mismatch between the ligand shell and the inorganic core, according to which a larger dielectric confinement implies a larger optical transition probability. The experimental data, instead, clearly point to a ligand contribution to the QD density of states of the entire valence and conduction bands that is related to the π conjugation of the pendant moiety.[^18^, ^31^, ^59^] The ligand π and π* orbitals contribution to the density of states is however mediated by the binding group: in fact, the broadband absorption enhancement induced by benzenethiol derivatives is about twice than that induced by cinnamic acids and lower than that induced by selenophenol.

## Towards a Unified Description of the Ligands’ Contribution to the QD Electronic Structure

**Figure 8:**
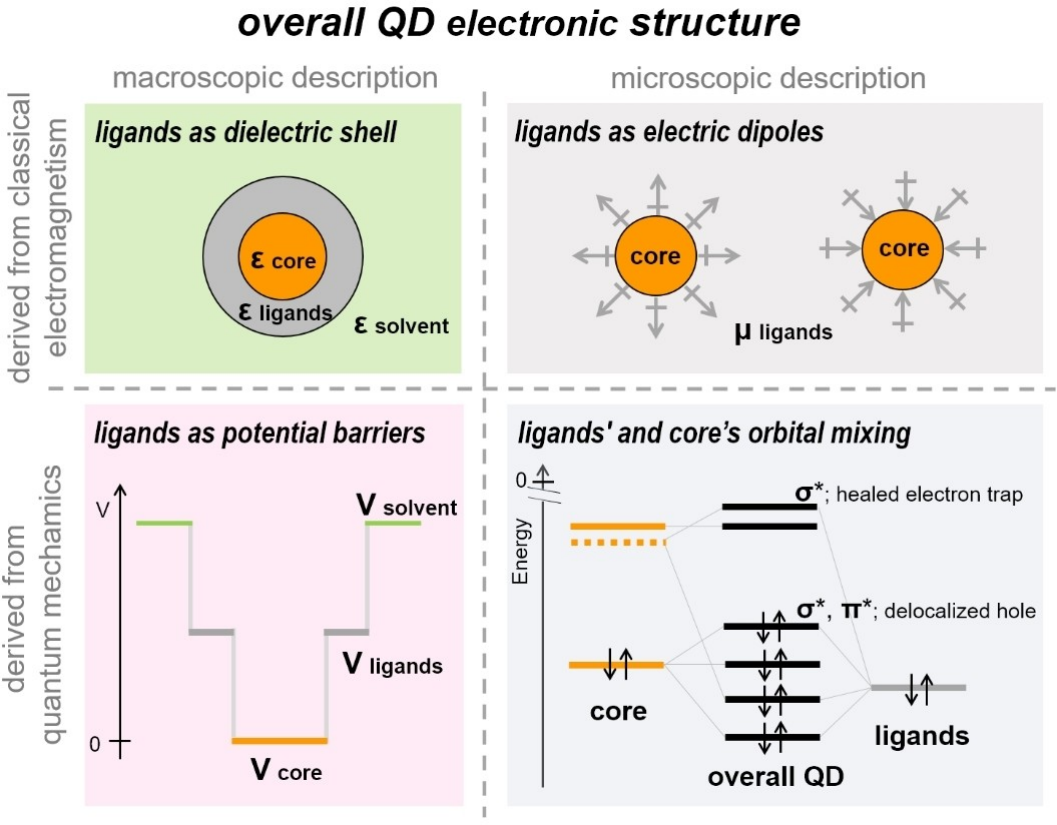
Sketch resuming the descriptions of the ligands at the QD surface by using electrostatic arguments either as a dielectric shell or as electric dipoles and by quantum mechanics as a potential energy barrier compared to the description based on the mixing of ligands’ and core's orbitals. The depiction of an eventual mixing of ligands’ lowest unoccupied orbitals with the unoccupied orbitals of the core and mixing of orbitals far from the band edges were omitted for clarity. Adapted with permission from ref. [3]; copyright 2020, American Chemical Society.

The abovementioned library of cinnamic acids was shown to induce, in concomitance with the broadband enhancement of the QD absorption, a shift of the QD energy levels as large as about 2 eV, which was mainly ascribed to the intrinsic dipole moment of the ligands.^[72]^ Indeed, dipoles that point toward (outward) the core decrease (increase) the overall QD ionization potential. Similar qualitative trends were observed for CdS and PbS QDs coordinated by benzenethiol derivatives.[^73^, ^74^] However, dipoles, despite representing a simple, effective description of the ligands inducing a shift of the QD energy levels,[^19^, ^72^, ^73^, ^74^] cannot explain the concomitant ligand‐induced broadband enhancement of the QD absorption. As already discussed for the changes of both the QD optical band gap and absorption coefficients, purely electrostatic arguments do not provide a comprehensive explanation of the effects exerted by the ligands on the overall QD electronic structure. Indeed, while the ligands and the core experience each other's electric field, the orbital mixing leads to delocalized states throughout the whole band structure. This explanation of the experimental data was backed up by density functional theory calculations of the electronic structure of metal‐rich, ligand‐coordinated QD model clusters.[^18^, ^31^] This explanation relies on the conception of the overall ligand/core adduct (the colloidal QD itself) as an indecomposable species and overcomes the above discussed limitations of conventional models that describe the ligands on the basis of mere electrostatic arguments (i. e., either as a dielectric shell or as electric dipoles) or as a potential energy barrier at the core boundaries (Figure 8).

The discussed ligand‐induced changes to the ionization potential, the optical band gap, and the absorption coefficients at all wavelengths of colloidal QDs have not, to date, been reported for lead halide perovskite nanocrystals. This may be related to the difficulties in avoiding the structural and morphological transformations of the halide perovskites upon ligand exchange and also to the sizes that are commonly attained by colloidal synthesis, yielding nanocrystals that experience a weak quantum confinement regime. However, it can be expected that reliable surface chemistry modification reactions on small, strongly quantum confined perovskite QDs^[75]^ may allow the observation of phenomena similar to those hereinabove discussed for metal chalcogenide QDs.

## Summary and Outlook

Surface chemistry is widely recognized as of capital importance to the science and technology of nanomaterials at large. Colloidal QDs represent an ideal platform to study the impact of the surface chemistry on the ground state electronic structure of nanomaterials since it was shown to induce large changes to the QD ionization potential, optical band gap, and absorption coefficients at all wavelengths. As the research on colloidal QDs moves toward an atomic level description of their chemical structure that also includes the surface species, the description of their electronic structure analogously progresses toward a comprehensive representation that considers the inherent electronic coupling of the inorganic core with the surface species. The description and explanation of the colloidal QD electronic structure at the ground state that includes the surface species is mandatory to control exciton dynamics (recombination, transfer, dissociation), which presides over the potential application of the colloidal QDs as light harvesting materials (in luminescence downconverters, photovoltaic cells, photocatalytic systems, among others).

## Conflict of interest

The authors declare no conflict of interest.

## Biographical Information

*Carlo Giansante studied chemistry at the Università di Bologna dealing with the photophysics and electrochemistry of discrete supramolecular systems. He also worked on the non‐linear optical properties of conjugated polymers at the University of California, Santa Barbara. He then studied, at the Université de Bordeaux, self‐assembled organic nanostructures and their photophysics down to the single molecule level. He's now at the Consiglio Nazionale delle Ricerche, Istituto di Nanotecnologia in Lecce where he established a research line on nanoscale surfaces and interfaces mostly relying on colloidal inorganic nanocrystals*.